# EEG spectral exponent as a synthetic index for the longitudinal assessment of stroke recovery

**DOI:** 10.1016/j.clinph.2022.02.022

**Published:** 2022-05

**Authors:** J. Lanzone, M.A. Colombo, S. Sarasso, F. Zappasodi, M. Rosanova, M. Massimini, V. Di Lazzaro, G. Assenza

**Affiliations:** a Neurorehabilitation Department, IRCCS Istituti Clinici Scientifici Salvatore Maugeri di Milano, 20138 Milan, Italy; b Department of Biomedical and Clinical Sciences “L. Sacco”, Università degli Studi di Milano, Italy; c Department of Neuroscience, Imaging and Clinical Sciences and Institute for Advanced Biomedical Technologies, 'Gabriele d'Annunzio University, Chieti, Italy; d IRCCS, Fondazione Don Carlo Gnocchi, 20148 Milan, Italy; e Università Campus-Biomedico di Roma, Neurology and Neurophysiology Unit, Italy

**Keywords:** Stroke, EEG, qEEG, Rehabilitation

## Abstract

**Objective:**

Quantitative Electroencephalography (qEEG) can capture changes in brain activity following stroke. qEEG metrics traditionally focus on oscillatory activity, however recent findings highlight the importance of aperiodic (power-law) structure in characterizing pathological brain states. We assessed neurophysiological alterations and recovery after mono-hemispheric stroke by means of the Spectral Exponent (SE), a metric that reflects EEG slowing and quantifies the power-law decay of the EEG Power Spectral Density (PSD).

**Methods:**

Eighteen patients (n = 18) with mild to moderate mono-hemispheric Middle Cerebral Artery (MCA) ischaemic stroke were retrospectively enrolled for this study. Patients underwent EEG recording in the sub-acute phase (T0) and after 2 months of physical rehabilitation (T1). Sixteen healthy controls (HC; n = 16) matched by age and sex were enrolled as a normative group. SE values and narrow-band PSD were estimated for each recording. We compared SE and band-power between patients and HC, and between the affected (AH) and unaffected hemisphere (UH) at T0 and T1 in patients.

**Results:**

At T0, stroke patients showed significantly more negative SE values than HC (p = 0.003), reflecting broad-band EEG slowing. Most important, in patients SE over the AH was consistently more negative compared to the UH and showed a renormalization at T1. This SE renormalization significantly correlated with National Institute of Health Stroke Scale (NIHSS) improvement (R = 0.63, p = 0.005).

**Conclusions:**

SE is a reliable readout of the neurophysiological and clinical alterations occurring after an ischaemic cortical lesion.

**Significance:**

SE promise to be a robust method to monitor and predict patients’ functional outcome.

## Highlights

• The Spectral Exponent (SE) indexes power-law features of the resting EEG in stroke patients.
• SE is consistently steeper in the affected hemisphere of patients after middle cerebral artery stroke.
• SE is linked to clinical status and seems to be a good predictor of clinical outcome.

## 1 Introduction

Stroke is a leading cause of disability and constitutes a heavy burden for healthcare systems (Gorelick, 2019). Monitoring brain activity with electroencephalography (EEG) could be a widely accessible and versatile instrument to aid the care of chronic and acute stroke patients. Indeed, given its ease of use, EEG is particularly suited for long-term monitoring of critical patients (i.e., intensive care units) as well as for the longitudinal assessment of patients with chronic stroke. In this context, quantitative EEG measures, such as ‘classical’ narrow-band Power Spectral Density (PSD), show a good correlation with stroke outcome (Finnigan and van Putten, 2013, Finnigan et al., 2008, 2007) and the literature is rich of reports on how stroke affects the power of different EEG frequency bands (Finnigan et al., 2016, Finnigan et al., 2008, Finnigan and van Putten, 2013). Typically, the power of lower frequencies (delta, theta) increases, while the power of higher frequencies (alpha, beta) decreases (van Wijngaarden et al., 2016). However, narrow-band power measures (e.g. delta power) conflate the periodic and aperiodic features of the signal (Donoghue et al., 2020a, Gao, 2016, Haller et al., 2018). Indeed, band-power measures of the EEG do not take into account the aperiodic 1/f-like component (He et al., 2010)--that largely dominates the EEG PSD. This 1/f-like shape is a general property of brain dynamics, consistent across different spatial scales, from single neurons (Destexhe et al., 2003), to meso/macro scale oscillations (Ciuciu et al., 2012, Dehghani et al., 2010, He, 2014), and is found in the decaying shape of the EEG’s PSD. The spectral exponent (SE) measures the steepness of the decay of the PSD background, relying on its 1/f-like structure. Specifically, it is computed as the slope of the PSD, in log-log coordinates, once the bias due to oscillatory peaks is minimized (Colombo et al., 2019, Donoghue et al., 2020b, Wen and Liu, 2016).

The SE is closely related to the clinical concept of EEG slowing, given that a slower EEG entails a steeper PSD decay. Additionally, the ratio between slow and fast frequencies, often used as a quantitative measure for stroke assessment (Finnigan et al., 2016, 2008, 2007), is directly affected by underlying changes in the PSD decay. EEG slowing is commonly observed after stroke and has been known from a clinical perspective for a long time (Giaquinto et al., 1994). More recently EEG slowing has been described also in qEEG studies, showing increase in delta activity or delta/alpha ratio (Finnigan et al., 2016, Finnigan and van Putten, 2013). EEG changes after stroke seem to be more prominent after large cortical ischemia (Fanciullacci et al., 2017) and have been related with the clinical status of the patient in different studies (Assenza et al., 2013, Saes et al., 2019, Zappasodi et al., 2017, 2019b). We propose that these EEG alterations could be also considered as the result of an overall change of the PSD shape, rather than changes in narrow-band power, thus justifying the use of a comprehensive measure such as the SE.

The slope of the PSD decay has also been linked to the neuronal balance between excitation and inhibition (E/I) (Gao et al., 2017). As described by Gao and colleagues, when the contribution of the inhibitory population to local field potentials (LFP) was increased, the decay of the PSD in simulated local field potentials becomes steeper (Gao et al., 2017). This effect was confirmed in various experimental set-ups, studying the spatial and temporal modulations of the E/I in rodents, and under anesthesia in macaques.

Furthermore, the PSD decay in humans resulted to be steeper in conditions typically related with increased inhibition, such as NREM sleep (He et al., 2010, Miskovic et al., 2019, Shen et al., 2003) or general anesthesia (Colombo et al., 2019), when compared to wakefulness – as indexed by more negative SE values (Freeman, 2006, Freeman and Zhai, 2009, Miskovic et al., 2019, Pereda et al., 1998). Therefore, the use of SE is gaining popularity also in the study of neurologic conditions (Belova et al., 2021, Molina et al., 2020, Pani et al., 2021, Wilkinson and Nelson, 2021).

Interestingly, more negative SE values (paralleled by reduced E/I ratio) were found over the affected hemisphere in rat models of stroke (Leemburg et al., 2018). Since the SE captures EEG slowing and is sensitive to alterations in the E/I balance, we expect it to be a reliable measure of the neurophysiological alterations in stroke. Accordingly, shifts in E/I ratio are a paramount feature in the neurophysiology of stroke (Carmichael, 2012) and several studies using Transcranial Magnetic Stimulation (TMS) consistently show altered excitability over the affected hemisphere (Malcolm et al., 2015, McDonnell and Stinear, 2017).

Given the above, we hypothesize that the SE, an index of the broad-band aperiodic EEG activity, could be a useful estimate of the state of cortical circuits in stroke patients, and be predictive of post-stroke functional outcome.

Here we test the SE on a longitudinal EEG data set and assess its sensitivity to the effects of both acute and chronic stroke, and its modulation following 1 month of standard physical rehabilitation. We show that the SE of EEG is an informative read-out of ischaemic brain lesions as it is intrinsically linked to the neurophysiological fingerprint of stroke.

## 2 Materials and methods

For the purpose, of this study, we retrospectively analysed EEG recordings from 23 patients (14 males, age 72 ± 9.5 y.o. mean ± sd, 22right-handed) diagnosed with mono-hemispheric stroke (14 left hemisphere stroke, 13 strokes with cortical involvement) in the territory of the middle cerebral artery (MCA) and 16 Healthy Controls (HC) matched for age and sex (7 males, age 68 ± 10 y.o. mean ± sd, 15 right-handed). EEGs were recorded at Campus Bio-medico University of Rome and “Casa di cura San Raffaele Cassino”. Written informed consent, of the protocol approved by the local ethical committee, was obtained from all participants. All procedures were in accordance with the ethical standards with the 1964 Declaration of Helsinki and its later amendments. Each patient had EEG recorded at T0 in the acute phase (a median of 6 days, Inter Quartile Range (IQR) 4|10 days, after the event), and at T1 after about 2 months, at the end of rehabilitation (median 77 days, IQR 62|88 days after the event).

Between T0 and T1 time points all patients underwent a standardized, 1 month long, protocol of rehabilitation based on physical therapy. EEG recordings consisted of 10 min resting, waking EEG with eyes closed.

Clinical inclusion criteria were: **i)** First ever ischemic stroke of MCA territory confirmed by MRI; ii) Evidence of motor/sensory deficit of the upper limb as assessed by a neurologist.

Exclusion criteria were: **i)** clinical history of previous stroke. **ii)** If patients could not comply with EEG recording. **iii)** If there was clinical/radiological evidence of acute bilateral involvement, brain haemorrhage, dementia or other neurodegenerative diseases such as Parkinson’s disease.

Exclusion criteria from EEG analysis were: 1) more than 1 bad channel (out of 19) in the EEG recording 2) lack of at least 180 s free from artefacts (jumps in the EEG signal, head movement, electrode pop) 3) lack of at least 180 seconds of closed eyes wakefulness.

If any of the above criteria occurred in the EEG at T0 or at T1 the patient was excluded from the study. Average data length was 193 ± 7 s (mean ± sd), and no patient had to be excluded from the study.

Neurological status was assessed at both T0 and T1 using the National Institute of Health Stroke Scale (NIHSS) [34]. NIHSS is an 11 items scale used in the assessment of clinical impairment related with stroke, range 0–42, with higher values reflecting more severe damages. Patients underwent 1.5 Tesla MRI scan at T0 as part of the diagnostic work-up, and the affected hemisphere was determined according to cerebral imaging and clinical findings. Lesion locations were subdivided into cortical (cortical or cortical/subcortical involvement) and purely sub-cortical (no clinical/radiologic evidence of cortical involvement) according to MRI findings. Supplementary Table 1 shows the NIHSS scores and the location of the ischaemic lesion in our patients.

### 2.1 EEG recordings

Both groups of MCA patients and HC underwent 19 channels EEG recording with standard 10–20 montage we show a representative process (Nuwer et al., 1998). 10 min of eyes closed EEG were recorded. Since the SE is minimally influenced by local spectral peaks (e.g. alpha peak) (Colombo et al., 2019), the eyes closed condition was chosen to help minimizing eye movements, as well as scalp muscle artifacts during the recording. The awake state of subjects was continuously monitored by EEG inspection, to exclude sleepiness and drowsiness. Signals were recorded with a 32 channel Micromed system (SystemPlus software; Micromed, Mogliano Veneto, IT) sampled at 256 Hz (16 bit A/D conversion) and online referenced to an electrode placed on the digitally linked mastoid. Contact impedance was kept below 5KΩ. Data was exported in EDF format for further analysis.

### 2.2 Pipeline to analyse the EEG signal

EEG recordings were analysed using MATLAB© native code and the FieldTrip toolbox (Oostenveld et al., 2011). The pipeline for the analysis focused on eliminating the major confounders of SE. On the one hand, eye movements and EEG signal artefactual deflections may increase delta power causing an artificially steeper slope, and on the other hand, intense muscular activity may increase power in the high frequency range, causing an artificially shallower slope.

**Fig. 1:**
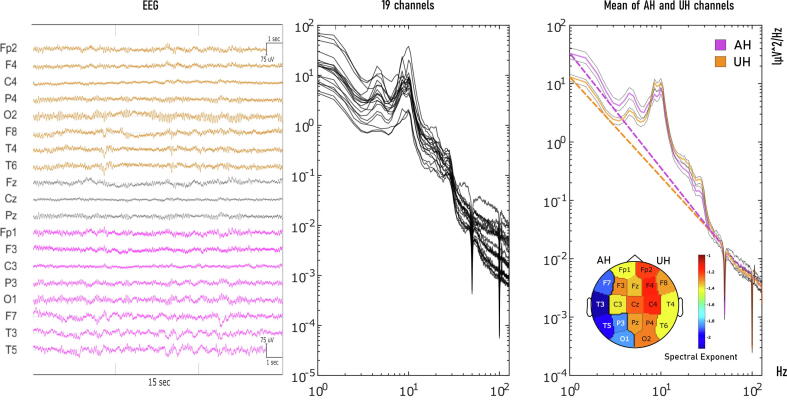
The spectral exponent reflects the decay of the PSD across a broad range of frequencies. Left panel: representative 15-s EEG traces for one patient (patient #17; left parietal stroke) recorded at T0. All channels with average reference are shown (AH in purple; UH in orange). Middle panel: PSD of the same EEG recording estimated (Welch) for all 19 channels after average referencing. Right panel: average PSD across AH channels (Purple), UH channels (Orange). The dashed lines represent the power-law fitted to the 1–40 Hz frequency range and the slope of these lines corresponds to the SE value. In the lower right corner, we show the topographic representation of SE values for each channel. SE = Spectral Exponent; AH = Affected Hemisphere; UH = Unaffected Hemisphere, PSD = Power Spectral Density.

EEG were imported from EDF format with acquisition reference and additional information regarding the channel location was added to the EEG structure. Long-range linear trends in the time-series were removed. Outliers among EEG channels were recognized using Z-score deviation over channels; the rejection threshold was set by visual inspection of each recording. Single bad channels were interpolated (nearest neighbour). Data was filtered with an IIR high-pass (5th order Butterworth filter with a 0.5 Hz cut-off) and a notch filter centred at 50 Hz. Scalp muscle artifacts were removed using Canonical Correlation Analysis (CCA), as described by De Clercq et al (De Clercq et al., 2006). Temporal autocorrelations in the EEG components were calculated with a sliding window of 10 s and 1 data point delay (1/256 Hz = 3.9 ms), components were ordered from the most autocorrelated to the least autocorrelated. Components with low autocorrelation (and thus likely of muscular origin) were removed from each window. The rejection threshold for autocorrelation was set at 0.7; this threshold was heuristically determined by inspection of time-series, PSD and scalp topography of each component. Data were then re-referenced to average reference. Independent Component Analysis (ICA) was performed and only components with clear ocular artifact were rejected by visual inspection of component’s topography, time-frequency and time series (average number of rejected components across EEG recordings: 1 ± 0.7). Signals were visually reviewed and residual eye movements or EEG jumps were excluded from PSD calculation. The PSD was estimated using Welch’s method (2 s window, 50 % overlap). SE of the 1–40 Hz-range was estimated for each pre-processed EEG channel. The code to estimate the SE is openly available online (https://github.com/milecombo/spectralExponent) and thoroughly explained in Colombo et al [26]. The topography of the SE value for each channel was plotted for visualization. In Fig. 1 we show a representative process of SE calculation; further insight in SE is shown in Supplementary Fig. 1. Delta (1–4 Hz), Theta (4–8 Hz), Alpha (8–12 Hz) and Beta (13–20 Hz) narrow-band power was also calculated for each EEG channel in order to compare SE with other quantitative measures derived from the PSD. Specifically, for each frequency band we computed the absolute band power as well as the normalized relative power (band power/total power). In addition, we computed Delta/Alpha ratio and Delta + Theta/Alpha + Beta (DTABR) ratio given their common application in the EEG stroke evaluation (Shreve et al., 2019).

### 2.3 Statistical analysis

The SE of PSD in the 1–40 Hz range results in a single value for each EEG channel. Data normality was checked using a quantile-quantile method. To test overall difference in SE between stroke patients and HC, we first compared the between-groups average SE across the 19 channels in each EEG recording at T0 and at T1, using unpaired t-test.

Then, we assessed the inter-hemispheric effect of the lesion on SE for the stroke patient group. We discarded midline EEG channels (‘Fz’;’Cz’;’Pz’) and sorted the remaining 16 channels into the affected hemisphere and unaffected hemisphere (AH and UH) for each patient individually, according to neuroimaging evaluation and clinical features. The mean SE value of the AH and of the UH was calculated. A similar approach was used for HC to assess SE differences between the right and the left hemisphere.

We hereby employ-four condition labels, to index both the lesion-side and the time point: AHT0 (affected hemisphere, time point 0); UHT0 (unaffected hemisphere, time point 0); AHT1 (affected hemisphere, time point 1); UHT1 (unaffected hemisphere, time point 1). A general linear model was built in R©(Bates et al., 2015, p. 4) in order to assess the main effects and their interactions. Specifically, a two-way repeated measures ANOVA was built, with the SE as dependent variable, hemisphere as within-subjects factor (levels: AH and UH), and time as within-subject repetition factor (levels: T0 and T1). Greenhouse-Geisser correction was used in the event of sphericity violation. Post-Hoc tests were performed using FDR correction for multiple comparisons. ANOVA assumptions were verified by quantile-quantile plotting and residual plot.

In addition, to verify the recovery of inter-hemispheric differences, the difference between the SE of the AH and UH was calculated for both T0 and T1 (SE[AH]- SE[UH]) for each patient, and a t-test against 0-mean assessed its change from T0 to T1.

The role of cortical vs subcortical lesions on SE was further assessed by means of unpaired t-test, comparing patients with cortical vs purely subcortical lesions.

Pearson’s correlation of the NIHSS scores was calculated with the SE values in the AH and UH, as well as with the AH-UH difference, for T0 and T1. In addition, we computed the effective recovery rate (ER)(Zappasodi et al., 2019a), a dynamic measure of patients’ functional recovery, and also assessed its correlation with the SE at both timepoints. ER is calculated as the percentage of the occurred improvement with respect to the total possible improvement, considering that NIHSS = 0 corresponds to the absence of clinical symptoms:.

$$\mathrm{ER}=100*\frac{NIHSSatT0-NIHSSatT1}{NIHSSatT0-0}$$

Finally, we performed an exploratory analysis of other narrow-band PSD metrics: we tested differences between AH and UH and T0 and T1 in absolute power and normalized relative power for all frequency bands, as well as in Delta/Alpha and Theta/Alpha ratios using Wilcoxon signed-rank tests. These exploratory comparisons are presented without any form of correction to maximize sensitivity. SE exponent statistics are reported as mean ± standard deviation (sd). Mean difference between groups and bootstrap 95 % Confidence Interval (CI95) were calculated. PSD metrics data are reported as median with Inter Quartile Range (IQR). For all statistical comparisons, the alpha level of significance was set at 0.05.

## 3 Results

### 3.1 Study population

5 patients matched the exclusion criteria due to poor compliance to EEG recording and/or poor quality of EEG signal and were thus not included. After removing these patients, our population comprised 18 patients (mean age 71.8 ± 8.9 years, 10 males, 17 right-handed, 11 left hemisphere stroke, 12 with cortical involvement) and 16 Healthy Controls (HC) participants (7 males, age 68 ± 10 y.o. mean ± sd, 15 right-handed).

### 3.2 Clinical features

None of the patient enrolled underwent systemic thrombolysis, since they arrived at the emergency room out of time, according to the latest guidelines for stroke management (Powers et al., 2019). NIHSS scores at T0 (median 5, IQR 2|9) were significantly higher (T(19) = 2,54p = 0.02) than at T1 (median 2, IQR 0.25|2). This functional recovery was also quantified by means of the ER (median 0.634, IQR 0.34|0.96).

### 3.3 Spectral Exponent in stroke patients vs healthy controls

Patients at T0 had a significantly steeper PSD (more negative SE values) compared to HC subjects (comparisons were made on the mean of all 19 channels from each subject),.

**HC:** SE:-1.12 ± 0.24; **PatientsT0:** SE:-1.48 ± 0.27 mean ± sd; Mean difference 0,39 ± 0,36; CI95 0,19|0,60, **T test**: T = -3.24, p = 0.0029.

The same comparison for patients at T1 was only marginally significant (**PatientsT1:** SE −1.33 ± 0.30; mean ± sd; Mean difference 0,24 ± 0,40; CI95 0,02|0,60, **T test**: T = -1.54p = 0.05), suggesting an overall renormalization of SE values over time in our patients group. As expected, HC did not show any significant inter-hemispheric asymmetry in the SE (**SE-Left** −1.13 ± 0.22 mean ± sd; **SE-Right** = -1.10 ± 0.25 mean ± sd; Mean difference 0,14 ± 0,30; CI95 −0,12|0,20, **T test:**T = -0.28, p-value = 0.77).

### 3.4 Effects of lesions and time on SE in stroke patients.

**Fig. 2:**
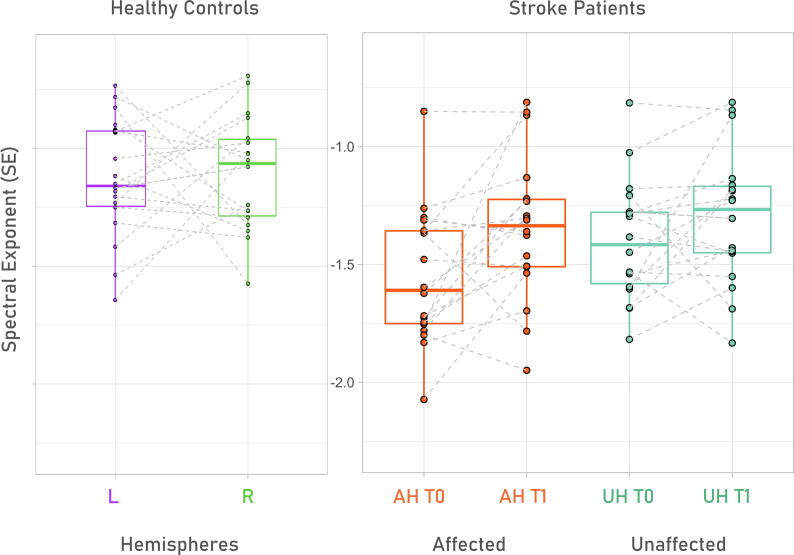
The SE is steep in patient with stroke compared to healthy control values. The affected hemisphere shows more negative values than the unaffected one, time recovers in part this effect. In the left panel we show normative SE values from healthy controls’ left (L, purple) and right (R, green) hemispheres. In the right panel we plot the distribution of SE affected (AH, orange) and unaffected (UH, blue) hemisphere both at T0 and T1, as boxplots with overlaying case distribution. Dotted lines connect T0 and T1 of each patient. SE = Spectral Exponent; AH = Affected Hemisphere; UH = Unaffected Hemisphere; L = Left; R = Right; T0 = 6 days after acute event (median); T1 = 2 months after acute event (median).

**Fig. 3:**
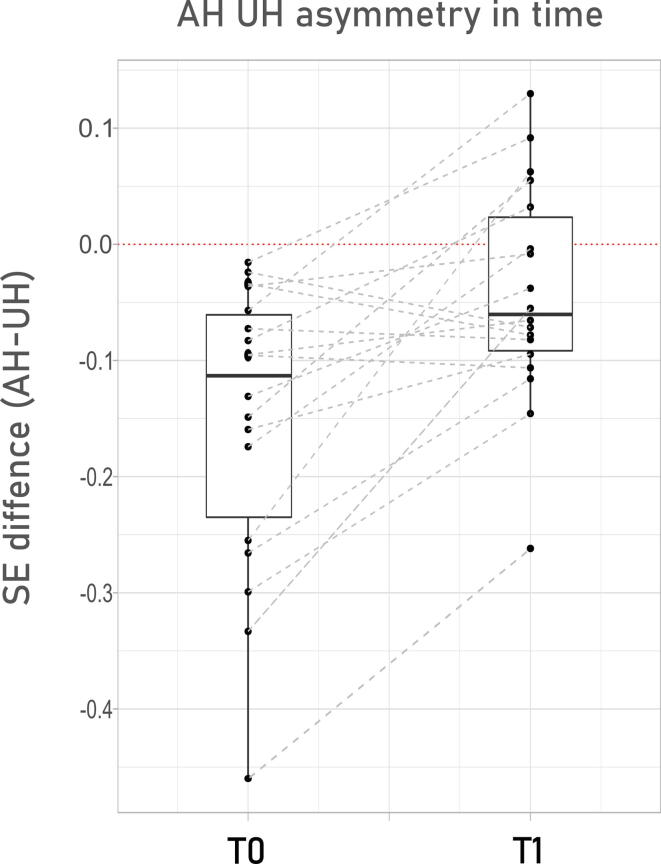
Asymmetry between the affected and unaffected hemisphere recovers with time. We show how hemispheric SE asymmetry (AH-UH) is always unbalances towards steeper valued in the AH at T0, and how this effect recovers in time, with most cases improving asymmetry. Dotted red line signs ideal symmetry. SE = Spectral Exponent; AH = Affected Hemisphere; UH = Unaffected Hemisphere; T0 = 6 days after acute event (median); T1 = 2 months after acute event (median).

Fig. 2 displays the two-way repeated measures ANOVA which showed significant main effects of both time (F [1,17] = 4.77, p = 0.043, η^2^ = 0.196) and hemisphere (F [1,17] = 16.88p = 0.001, η^2^ = 0.09) in the patients’ group. A significant interaction between time and hemisphere factors was also found (F [1,17] = 18.64, p = 0.0004, η^2^ = 0.02). Fig. 3 shows how hemispheric asymmetry changes in time.

Post-hoc analysis (FDR-corrected for multiple comparisons) evidenced significant SE differences in the following comparisons.

Inter-hemispheric differences at T0:.

AHT0 mean SE = -1.55 ± 0.289; UHT0 mean SE = -1.40 ± 0.254, mean ± sd;.

Mean difference −0,15 ± 0,12, CI95 −0,21|-0,09; T = 5.19p = 0.0001;.

Change in the affected hemisphere over time:.

AHT0 mean SE = -1.55 ± 0.289 vs AHT1 mean SE = -1.34 ± 0.309, mean ± sd;.

Mean diffefence −0,20 ± 0,29, CI95 −0,33|-0,05; T = 2.74p = 0.003;.

Affected hemisphere at T0 against its contralateral at T1:.

AHT0 mean SE = -1.55 ± 0.289 vs UHT1 mean SE:-1.30 ± 0.285, mean ± sd;.

Mean difference −0,25 ± 0,22, CI95 −0,42|-0,11; T = 3.41p = 0.001.

Overall, these results show the occurrence of a steeper PSD slope over the affected hemisphere that renormalizes over time. These findings are further supported by the significant reduction in the inter-hemispheric difference (SE[AH]- SE[UH]) between T0 and T1. (**T0 AH-UH mean SE =** -0.1522 ± 0.1244,**T1 AH-UH mean SE =** -0.0567 ± 0.1243 mean ± sd; Mean difference −0,105 ± 0,101, CI95 −0,15|-0,05; **T test**: T = -3.6847, p = 0.0018).

The type of lesion did not affect changes in the SE, since we did not find significant differences in SE when comparing SE between cortical (12 subjects) and non-cortical (6 subjects) lesions at T0 and at T1, both in the AH and in the UH (all P > 0.175 after correction).

### 3.5 SE **correlation** with clinical indicators.

Exploratory correlations of the SE with the NIHSS clinical scale and ER were performed across time-points in the affected and unaffected hemispheres. Results are shown in Table 1.

**Table 1:** **Spectral exponent allows to predict functional recovery.** The table shows correlation between SE values and clinical scores (un-corrected p-values). Significant values are highlighted in grey, significant findings after Bonferroni correction are highlighted in light blue.

	**NIHSS T0**	**NIHSS T1**	**ER**
**AH T0**	r = -0,31;p = n.s.;CI95 −0,18|0,67	r = -0,48;p = 0,04;CI95 0,01|0,72	r = 0,63;p = 0,004; CI95 0,23|0,84
**UH T0**	r = -0,25;p = n.s.;CI95 −0,24|0,64	r = -0,41;p = n.s.; CI95 −0,067|0,73	r = 0,60;p = 0,008;CI95 0,18|0,83
**AH T1**	r = -0,48;p = 0,04;CI95 0,02|0,77	r = -0,70;p = 0,001;CI95 0,35|0,88	r = 0,58;p = 0.01;CI95 0,16|0,82
**UH T1**	r = -0,44;p = n.s.;CI95 −0,03|0,75	r = -0,59;p = 0.01; CI95 0,17|0,82	r = 0,51;p = 0.03;CI95 0,05|0,78

SE = Spectral Exponent; AH = Affected Hemisphere; UH = Unaffected Hemisphere; NIHSS = National Institute of Health Stroke Scale; ER = Effective Recovery; T0 = 6 days after acute event (median); T1 = 2 months after acute event (median); CI95 = 95 % Confidence Interval.

**Fig. 4:**
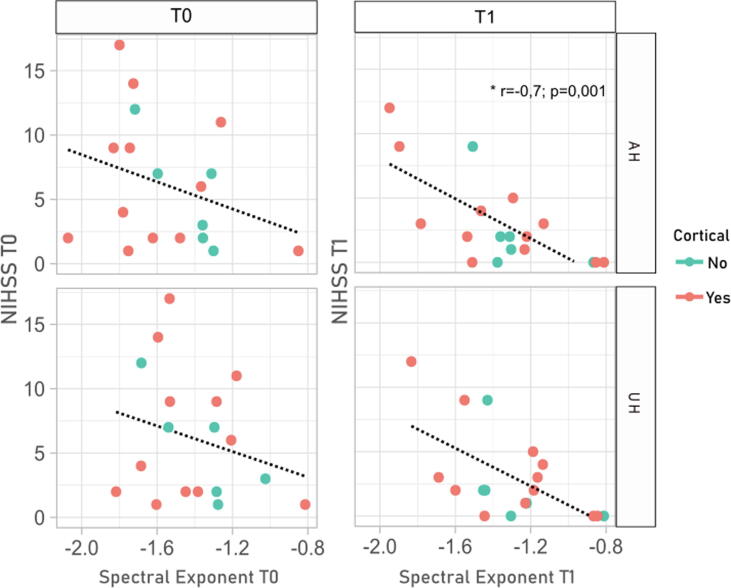
The SE is correlated with clinical current status. The figure shows the correlation between SE values in the AH and UH and NIHSS; here we show the relation of SE with concomitant values of NIHSS (T0 with T0, T1 with T1). Lesions with subcortical or cortical involvement are color-coded. Significant correlation after multiple comparison correction is noted, full correlation matrix is shown in Table 1. SE = Spectral Exponent; AH = Affected Hemisphere; UH = Unaffected Hemisphere; NIHSS = National Institute of Health Stroke Scale; T0 = 6 days after acute event (median); T1 = 2 months after acute event (median).

**Fig. 5:**
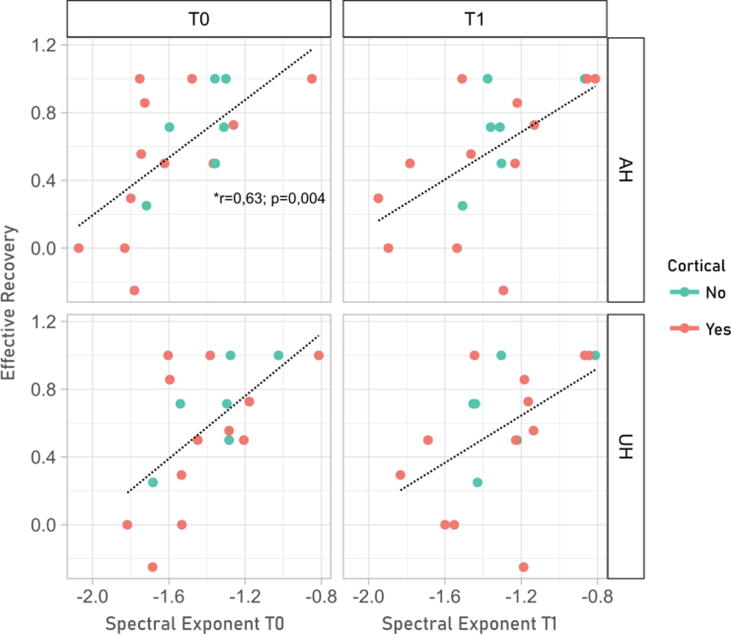
**The SE allows predicting post-stroke functional recovery.** The figure shows the correlation between SE values, in different hemispheres and across time points, with the effective recovery (ER) of patients. Lesions with and without cortical involvement are color-coded. Significant correlation after multiple comparison correction is noted, full correlation matrix is shown in Table 1. SE = Spectral Exponent; AH = Affected Hemisphere; UH = Unaffected Hemisphere, ER = Effective Recovery; T0 = 6 days after acute event (median); T1 = 2 months after acute event (median).

The SE showed good correlation with clinical outcomes. SE values at T0 showed a weaker correlation with NIHSS (both T0 and T1) compared to SE values at T1. Meanwhile, ER was always correlated with SE regardless of the hemisphere or timepoint, with the strongest correlation being with SE in the AH at T0. In Fig. 4 and Fig. 5 we show the relation between SE and clinical scores, highlighting a similar pattern across cortical and non-cortical lesions.

### 3.6 Conventional narrow-band power metrics.

In Supplementary Table 2 we show the results of multiple tests comparing differences between conventional narrowband metrics derived from the PSD: log transformed absolute power, relative power as well as Delta/Alpha ratio (DAR), Delta + Theta/Alpha + Beta ratio (DTABR) and Theta/Alpha ratio. Commonly used spectral indexes did not evidence differences between the AH and the UH, showing only trends for Delta power and Delta/Alpha ratio (P > 0.14). Significant differences (P < 0.05) between T0 and T1 PSD were evidenced, mainly in log transformed Beta and delta power, showing that from T0 to T1 there is decrease of delta power and increase of beta power. When using normalized measures, higher Relative Delta power was evident at T0 both in the AH and UH, while Relative Alpha and Relative Beta bands were found lower at T0 both over the AH and the UH. Also, DAR was significantly higher at T0 compared to T1 and also DTABR showed the same pattern.

## 4 Discussion

In this study, the spectral exponent identified the lesioned hemisphere in patients affected by MCA stroke, and its longitudinal changes paralleled the recovery of clinical impairment. Specifically, we showed that mono-hemispheric stroke is characterized by a steeper PSD decay, indexed by more negative global SE values, compared to HC, shortly after the insult (T0), as can be seen in the exemplary use case in Supplementary Fig. 2. Most importantly, the SE is associated with the side of injury, with invariably more negative values over the affected hemisphere compared to the contralesional one, across all patients. Further, we showed that due to a prominent SE renormalization over the affected hemisphere, both local and global SE values attain similar values to those of HC after 2 months (T1). Highlighting the clinical relevance of these findings, the degree of SE renormalization reflects the degree of functional recovery. Overall, this study proposes the SE as a reliable electrophysiological fingerprint of stroke.

### 4.1 The SE as a broad-band synthetic index

Previous literature on qEEG in stroke proved that after brain ischemia a significant change in Delta band spectral activity is recorded (Jiang et al., 2019, Shreve et al., 2019, Tecchio et al., 2006), particularly in cases with large cortical involvement (Fanciullacci et al., 2017). Additionally, many studies described a close concordance between the area of maximum Delta power and the ischaemic area as shown by brain imaging (CT and MRI), suggesting that Delta activity might localize the injured area (Ajčević et al., 2019, Fernández-Bouzas et al., 2000, Machado et al., 2004, Murri et al., 1998, Shreve et al., 2019, Wu et al., 2016).

Further improvement on the specificity of qEEG was warranted by the introduction of the Delta/Alpha ratio (DAR), which in many cases showed better clinical correlation than Delta power alone (Finnigan et al., 2016, Finnigan et al., 2008, Finnigan et al., 2007, Finnigan and van Putten, 2013). The DAR, which measures the ratio between slow and fast oscillations, coarsely reflects the overall PSD shape, which is quantified by the spectral exponent. However, the DAR might be vulnerable to increases or decreases of Alpha/Delta spectral peaks such as alpha activity in the occipital derivation (eyes open vs closed) or increased delta activity due to non-cerebral artifacts (eye movements). Moreover, in our population the DAR showed a lower correlation with the site of lesion and with the clinical evolution, as compared to SE.

We here propose the SE as a novel qEEG synthetic metric, able to evidence clinically relevant asymmetries in brain activity, while providing at the same time a robust and comprehensive representation of the PSD.

### 4.2 Neurophysiological determinants of the SE: E/I balance

Other than predicting functional outcome, the SE might help to better understand the pathophysiologic consequences of focal brain injury, by indexing alterations of the E/I balance.

Indeed, after ischaemic stroke the E/I balance in the lesioned area undergoes significant changes (Krnjević, 2008, Rabiller et al., 2015) that indirectly reflect on the EEG (Sohal and Rubenstein, 2019). During the hyperacute phase (first hours) neuronal depolarization and glutamate release cause transient hyperexcitability, thus increasing the E/I ratio (Fujioka Hiroshi et al., 2004, Schiene et al., 1996). Thereafter, during the sub-acute and chronic phases (after cell death has transpired), GABA currents are enhanced (Clarkson et al., 2010), causing a decrease in excitability with lower E/I ratio (Farrant and Nusser, 2005). These changes are thought to reflect on the EEG with increased low frequency activity and decreased fast frequency activity, which results in a steeper PSD shape (more negative SE values) (Leemburg et al., 2018, van Wijngaarden et al., 2016).

The increase in inhibition, taking place after stroke, is sustained by hyperactivation of tonic—but not phasic—GABA_A_ channels (Clarkson et al., 2010). We speculate that the increased tonic inhibition might be reflected in the aperiodic (power law) component of PSD rather than by its periodic features (Farrant and Nusser, 2005), more tightly linked to phasic inhibition (Rovo et al., 2014). Additionally, paired pulse stimulation protocols evidence paradoxically decreased GABAergic activity after stroke both in vitro (Domann et al., 1993) and in vivo (McDonnell and Stinear, 2017); this is possible since such protocols are sensitive to GABA_A_ phasic but not tonic activity (Carmichael, 2012). However, recently, TMS protocols on long lasting inhibition were able to confirm increased GABA tonic activity in stroke (Cirillo et al., 2020), further supporting this hypothesis.

A comparison between stroke patients using the SE and metrics derived from TMS-EEG measurements (Sarasso et al., 2020), which are in principle more sensitive to EEG changes related to phasic inhibition, could yield interesting insights regarding these phenomena.

Interestingly, the SE difference between the AH and UH yields values up to −0.3/-0.4 (absolute SE unit); Fig. 2. These changes are in the same order of magnitude of those induced by general anesthesia with Ketamine (-0.5), (Colombo et al., 2019). Thus, stroke causes a steepening in SE that is comparable with extreme conditions such as general anaesthesia.

Due to the previously mentioned limits of narrow band measures, other measures addressing the scale-free structure of EEG activity are growing in popularity, and lately methods for estimating the power-law behaviour of PSD have been implemented in popular toolboxes, such as Brainstorm (Haller et al., 2018). Independently from other scale free measures, SE has the advantage of being simple to interpret, fast to compute and, most importantly, neurophysiologically grounded to the idea that the E/I balance shapes the overall PSD decay (Gao et al., 2017, Miller et al., 2009). These features make it a putative candidate for reliably assessing the state of cortical circuits following focal brain injury at the patients’ bedside.

### 4.3 Potential clinical relevance of the SE

SE was positively correlated with the functional outcome of stroke patients, as assessed by the NIHSS scores, thus highlighting its potential as a predictor of the clinical consequences of stroke. This finding warrants the discussion of some interesting aspects.

SE in all hemispheres and timepoints correlates with the effective recovery rate (ER), and showed good correlation with NIHSS scores in the T1 timepoint. Notably, steeper slopes at T0 over the lesioned hemisphere were correlated with a lower degree of effective recovery, suggesting that broadband slowing in the lesioned hemisphere, will negatively affect the chances of improvements. This finding is particularly interesting and hints to a potential role of SE in predicting the functional outcome of stroke.

Ultimately, the SE seems to be a potential marker of clinical outcome and future work should compare it with other qEEG metrics as a predictor of common clinical indexes in stroke, such as the Rankin (Zappasodi et al., 2019b) or Fugl-Meyer (Saes et al., 2019) scale. Additionally, the commercialization of dry EEG caps (Fiedler et al., 2015) with short setup time, given that qEEG metrics are validated on such devices, will make EEG potentially viable even in acute and subacute settings, such as during thrombolysis (de Vos et al., 2008). Thus, in the near future quantitative EEG measures might have a significant role in predicting clinical outcome in stroke patients and implementing personalized approaches aimed at enhancing functional recovery, like non-invasive brain stimulation techniques (Di Pino et al., 2014, Takeuchi and Izumi, 2012) and EEG-guided robotic rehabilitation (Carvalho et al., 2019). Overall, SE provided a good correlation with NIHSS.

### 4.4 Cortical and subcortical stroke

We did not find significant SE or spectral differences when comparing cortical and non-cortical subgroups in our population. Recent findings by Sarasso et al (Sarasso et al., 2020)**,** using TMS-EEG, showed clear differences in TMS-evoked oscillatory activity between cortical and non-cortical lesions. This could suggest that SE addresses aperiodic features that are shared by cortical and subcortical lesions, while the two conditions have differences in the frequency domain that can be better appreciated with perturbation approaches.

Previous literature reports clear spectral changes in MCA cortical strokes (Fanciullacci et al., 2017), while similar evidence is less consistent for subcortical, lacunar and posterior circulation strokes (Macdonell et al., 1988). Further data on SE in various types of stroke should be collected, in order to confirm that the SE is sensitive also in cases of subcortical stroke, as it seems according to our data. In principle, while cortical lesions are known to have increased delta power, subcortical lesions do not consistently show this alteration. Some authors described reduced spontaneous EEG complexity in cases of subcortical stroke (Shen et al., 2003), which mostly relates to loss of fast activity. Reduced complexity could produce faint changes in the overall broad-band shape of PSD, that could be detected more easily by SE compared to other qEEG metrics.

## 5 Limitations

Our study presents some limitations that need to be addressed by future studies. Our sample size was relatively small and further studies on a larger population should be conducted to understand the sensitivity and specificity of SE in stroke. Patients enrolled had mild NIHSS scores,since we needed compliance to EEG recording most severe patients had to be excluded and this might be the cause of potential selection bias. Additionally NIHSS scale has some limitations as a metric bearing relation with qEEG (Marsh et al., 2016), being skewed towards the dominant hemisphere it might underestimate non-dominant hemisphere lesions.

The study design did not include appropriate imaging sequences for lesion volume calculation. As such, at this stage we could not explore the relation between lesion volume and SE. Also, the low density of EEG leads in our study does not allow for an assessment of the spatial resolution of SE with respect to the lesion location. Studying SE with high density EEG may allow to explore both local and long distance effects of ischemic lesions (Russo et al., 2021). Future works shall address these important questions by employing dense array EEG setups and appropriate MRI sequences.

## 6 Conclusions

In the present study we employed the SE as a synthetic qEEG measure, reflecting the power-law decay of the PSD background, to provide a neurophysiological fingerprint of stroke. The SE appears to be a good read-out of the neurophysiological state of cortical circuits following focal ischaemic lesions. Indeed, the SE showed inter-hemispheric differences consistent across all the patients in our sample, and a partial/complete renormalization over time in most patients, which followed the amount of functional recovery, as measured by clinical scales. The use of SE in stroke is coherent with the putative neurophysiological mechanisms triggered by brain ischaemia. Automated qEEG read out, compared with clinical interpretation, could be useful in continuous EEG monitoring and could offer quantitative rather than qualitative variables, that would help the construction of predictive outcome models. Simple yet effective measure as SE can be useful in the development of predictive models of outcome, or as a quick read-out for brain monitoring in ICU and could help guide future neurostimulation protocols.

## Funding

This work was supported by the European Union’s Horizon 2020 Framework Program for Research and Innovation under Specific Grant Agreement No. 945539 (Human Brain Project SGA3), by Fondazione Regionale per la Ricerca Biomedica (Regione Lombardia), Project ERAPERMED2019-101, GA779282, by the Tiny Blue Dot Foundation, by the Canadian Institute for Advanced Research (CIFAR).

## Declaration of Competing Interest

The authors declare that they have no known competing financial interests or personal relationships that could have appeared to influence the work reported in this paper.
