# Supplementary material for: EEG spectral exponent as a synthetic index for the longitudinal assessment of stroke recovery
Source: Clin Neurophysiol. 2022 May;137:92–101. doi: 10.1016/j.clinph.2022.02.022 (PMC9038588; doi:10.1016/j.clinph.2022.02.022)
Supplement: Supplementary figure 1 [file mmc1.pdf]

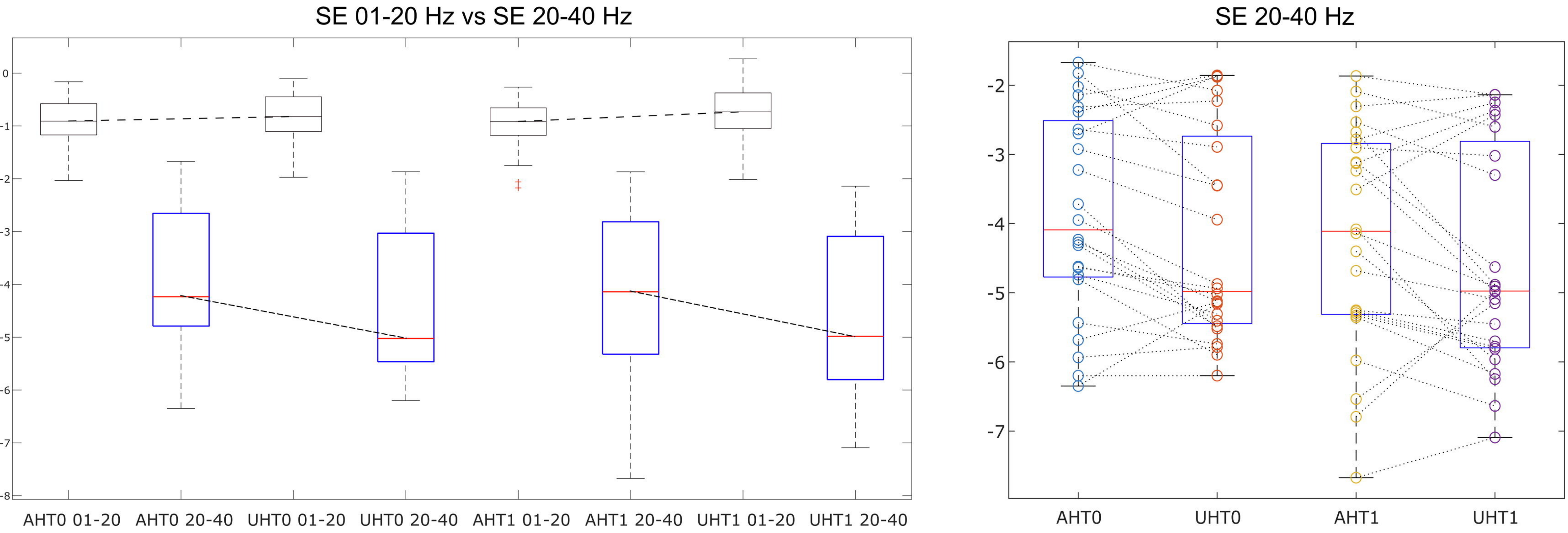

Supplementary Figure.1 Differences between 1-20 Hz and 20-40 Hz SE.

Conventionally, SE is calculated on the broad band (1-40 Hz range); however the PSD displays slightly different trends in the lower and higher frequency range (Colombo 2019). Here we further investigate whether the spectral exponent in two sub bands (1-20 Hz, 20-40 Hz) is differently affected by stroke. The 1-20 Hz SE resembles the 1-40 Hz SE, confirming that the PSD decay in the lower portion determines the main tendency of the overall broad-band spectrum, consistently with the notion of stroke-induced slowing. Interestingly, the results of the 20-40 Hz SE show an opposite trend, namely a flatter slope in perilesional areas, recovering over time. We speculate that this effect may result from a compensation of the excitatory drive recruited from the increased inhibitory drive, as indeed: Stronger inhibitory drive may recruit the excitatory drive, a concept known as excitatory/inhibitory balance. The excitatory synaptic activity (E) has a faster time constant than the inhibitory one (I), thus, while the first is reflected more by the higher frequency range of the PSD, the latter is reflected more by the lower frequency range. Together, the predominantly inhibitory synaptic activity shape the overall PSD decay, which is captured by the broad-band spectral exponent

AH=Affected Hemisphere; UH=Unaffected Hemisphere; T0=6 days after acute event(median); T1= two months after acute event (median)
