# Supplementary material for: EEG spectral exponent as a synthetic index for the longitudinal assessment of stroke recovery
Source: Clin Neurophysiol. 2022 May;137:92–101. doi: 10.1016/j.clinph.2022.02.022 (PMC9038588; doi:10.1016/j.clinph.2022.02.022)
Supplement: Supplementary figure 2 [file mmc2.pdf]

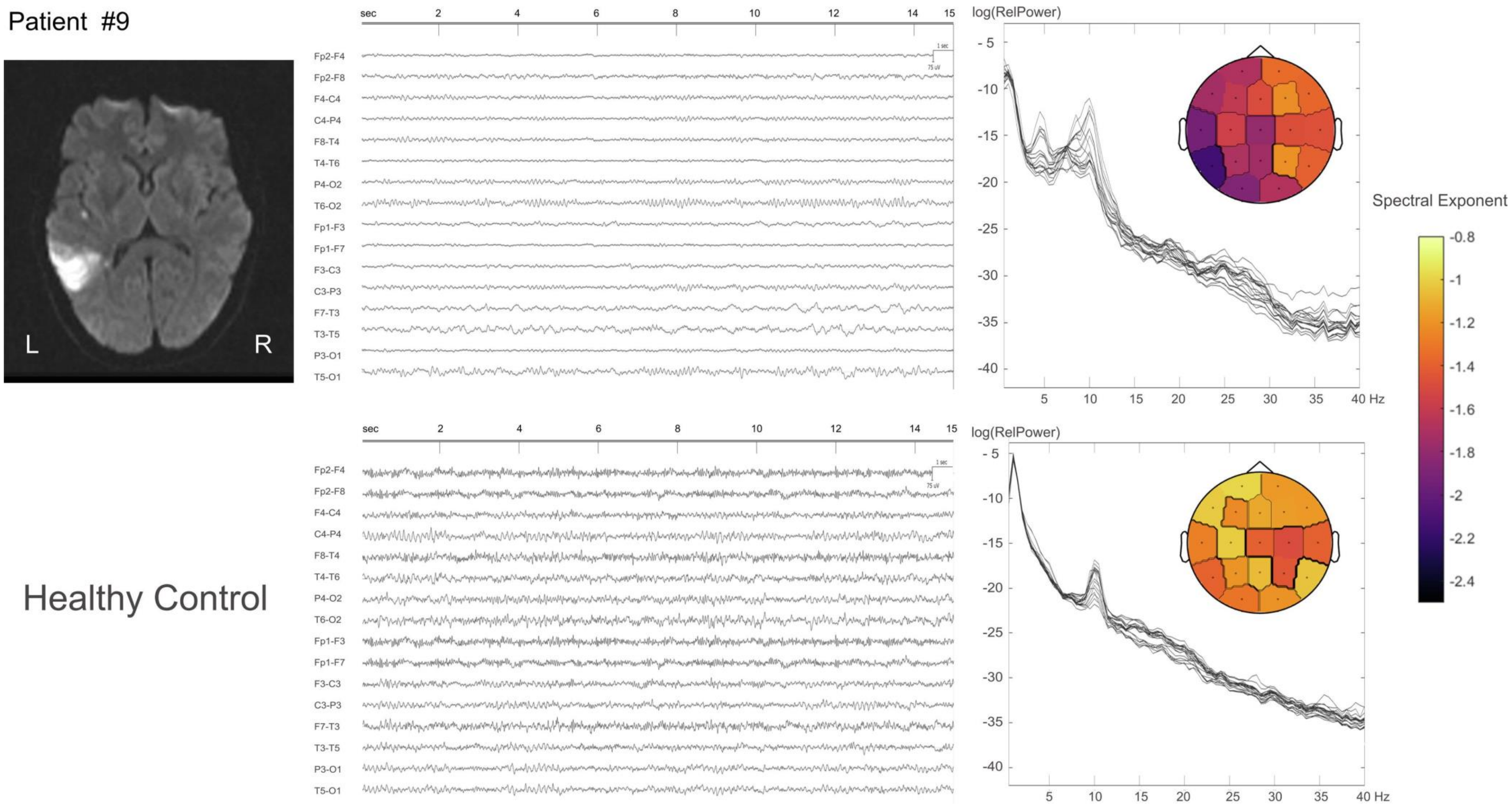


**Supplementary Figure.2 Spectral Exponent use case.**

In this figure we display EEG from patient #9 (left parietal lesion, as shown by Diffusion Weighted Imaging hyperintensity, image flipped for ease of understanding). We compare the EEG with a healthy control. In the EEG trace some slowing can be appreciated. We also plotted the Power Spectral Density (log-log scale) using relative power since it allows for more robust comparisons between subjects (less dependable on recording variables). In the PSD plot we can see that Patient #9 shows increased slow activity (theta and delta). Finally, we plotted the topography of Spectral Exponent calculation with shared scale, in patient#9 the topography shows that steeper SE values are surprisingly close to the lesion location on the MRI.

PSD=Power Spectral Density, MRI=Magnetic Resonance Imaging, L=Left, R=Right
